# The Role of Obesity, Inflammation and Sphingolipids in the Development of an Abdominal Aortic Aneurysm

**DOI:** 10.3390/nu14122438

**Published:** 2022-06-12

**Authors:** Jakub Okrzeja, Alicja Karwowska, Agnieszka Błachnio-Zabielska

**Affiliations:** Department of Hygiene, Epidemiology and Metabolic Disorders, Medical University of Bialystok, 15-089 Bialystok, Poland; okrzeja1@student.umb.edu.pl (J.O.); alicja.karwowska@umb.edu.pl (A.K.)

**Keywords:** abdominal aortic aneurysm, bioactive lipids, sphingolipids, sphingosine-1-phosphate, ceramides

## Abstract

Abdominal aortic aneurysm (AAA) is a local dilatation of the vessel equal to or exceeding 3 cm. It is a disease with a long preclinical period commonly without any symptoms in its initial stage. Undiagnosed for years, aneurysm often leads to death due to vessel rupture. The basis of AAA pathogenesis is inflammation, which is often associated with the excess of adipose tissue, especially perivascular adipose tissue, which synthesizes adipocytokines that exert a significant influence on the formation of aneurysms. Pro-inflammatory cytokines such as resistin, leptin, and TNFα have been shown to induce changes leading to the formation of aneurysms, while adiponectin is the only known compound that is secreted by adipose tissue and limits the development of aneurysms. However, in obesity, adiponectin levels decline. Moreover, inflammation is associated with an increase in the amount of macrophages infiltrating adipose tissue, which are the source of matrix metalloproteinases (MMP) involved in the degradation of the extracellular matrix, which are an important factor in the formation of aneurysms. In addition, an excess of body fat is associated with altered sphingolipid metabolism. It has been shown that among sphingolipids, there are compounds that play an opposite role in the cell: ceramide is a pro-apoptotic compound that mediates the development of inflammation, while sphingosine-1-phosphate exerts pro-proliferative and anti-inflammatory effects. It has been shown that the increase in the level of ceramide is associated with a decrease in the concentration of adiponectin, an increase in the concentration of TNFα, MMP-9 and reactive oxygen species (which contribute to the apoptosis of vascular smooth muscle cell). The available data indicate a potential relationship between obesity, inflammation and disturbed sphingolipid metabolism with the formation of aneurysms; therefore, the aim of this study was to systematize the current knowledge on the role of these factors in the pathogenesis of abdominal aortic aneurysm.

## 1. Introduction

Abdominal aortic aneurysm (AAA) is described as a localized aorta dilatation with a diameter of ≥3.0 cm. The main risk factors of AAA development include cigarette smoking and family history of AAA [1]. The coexistence of AAA with hypertension, atherosclerosis and unhealthy eating habits (considerable amount of fats and carbohydrates) leading to obesity is also frequently observed [1]. At the cellular and metabolic level, AAA is characterized by inflammation, vascular smooth muscle cell (VSMC) apoptosis, excessive reactive oxygen species (ROS) production and extracellular matrix degradation [2]. These factors cause weakening of the aortic wall and the inability to withstand the blood pressure forces, which results in progressive dilatation and can lead to rupture with an estimated mortality rate of 50–80% [3]. A balanced diet and active lifestyle appear to reduce the risk of an aortic dilatation [4]. The role of obesity as a risk factor for an abdominal aortic aneurysm is still poorly investigated, although available data suggest a link between AAA and obesity [5]. Obesity is defined as an excessive amount of body fat. In this state, the content of perivascular adipose tissue (PVAT) (adipose tissue which surrounds the blood vessels) also increases [6]. Moreover, obesity is often accompanied by moderate inflammation, as adipose tissue synthesizes and secretes many pro-inflammatory cytokines. Moreover, macrophages infiltrating adipose tissue, including PVAT, secrete a wide range of matrix metalloproteinases (MMPs) and are a key source of MMPs that are involved in extracellular matrix degradation, being an important factor in the formation of aneurysms [7].

**Figure 1:**
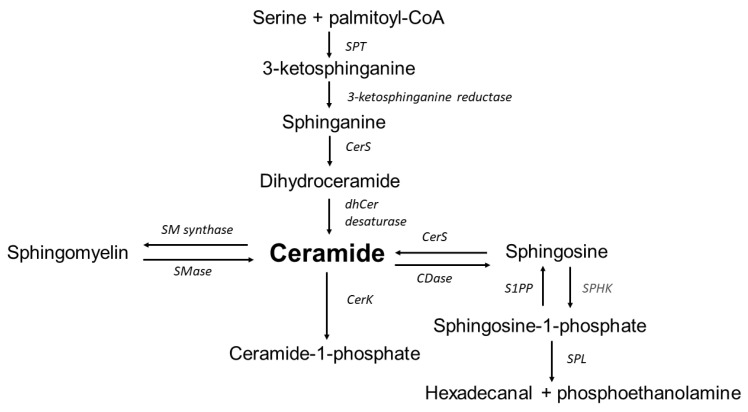
Overview of sphingolipid metabolism. SPT—serine palmitoyltransferase, CerS—ceramide synthase, dhCer desaturase—dihydroceramide desaturase, CerK—ceramide kinase, S1PP—sphingosine-1-phosphate phosphatase, SPHK—sphingosine kinase, SPL—sphingosine-1-phosphate lyase, CDase—ceramidase, SMase—sphingomyelinase, SM synthase—sphingomyelinase synthase. Ceramide is synthesized in the endoplasmic reticulum and transferred to the Golgi apparatus where it is subsequently transformed into complex sphingolipids. Ceramide is also produced by the degradation of sphingomyelin. Ceramide may be transformed into a variety of sphingolipid products, including sphingosine and S1P.

It has been demonstrated that the adipocytes of people with obesity increase not only the content of triacylglycerols but also biologically active lipids, including sphingolipids [6]. Sphingolipids are a large and functionally diverse group of compounds that play an important role in building membranes of eukaryotic cells and regulate important cellular processes such as proliferation, cell differentiation, apoptosis and inflammation, which are associated with the pathology of aneurysms [8]. This group of lipids includes compounds which, to some extent, play opposite functions in the cell, in particular ceramide and sphingosine-1-phosphate (S1P). Ceramide promotes apoptosis, growth arrest and inflammation, while sphingosine-1-phosphate acts as a mitogen, activating proliferation and angiogenesis but inhibiting apoptosis and inflammation. The diversity of cellular responses mediated by sphingolipids is due to the fact that these molecules regulate the activity of a wide variety of intracellular enzymes, including kinases, phosphatases and lipases [9]. It has been also repeatedly shown that ceramide induces the production of ROS and increases oxidative stress in many mammalian cells [10]. Altered levels of S1P and ceramides were reported in abdominal aortic cells (in cases with AAA) [6,11,12]. The inflammation state observed in the cells of the abdominal aorta may therefore be associated with disturbances in sphingolipid metabolism [13,14,15,16]. An overview of sphingolipid metabolism is presented in Figure 1.

The mechanism of pathogenesis of AAA is not entirely clear yet. Since the mortality rate associated with aneurysm rupture is extremely high, it is important to understand the mechanisms that lead to their formation. Literature data indicate that inflammation and disturbed sphingolipid metabolism associated with excess adipose tissue may be a significant risk factor for the development of AAA. Therefore, the aim of this review was to systematize the current knowledge on the role of obesity, inflammation and sphingolipids in the pathogenesis of an abdominal aortic aneurysm.

## 2. Materials and Methods

Life science databases, such as PubMed, Scopus, and Web of Science, were searched until April 2022 for the latest and the most interesting research on the role of obesity as well as the involvement of biologically active lipids in the pathogenesis of abdominal aortic aneurysm. The phrases searched for in connection with ‘abdominal aortic aneurysm’ included: ‘obesity’, ‘bioactive lipids’, ‘sphingosine-1-phosphate’, ‘ceramides’, ‘sphingolipids’, and ‘inflammation’. All the studies were in the English language.

## 3. Obesity as a Risk Factor of AAA

One of the potential risk factors for aneurysms is obesity. An unhealthy, high-fat diet causes hyperlipidemia, significantly burdens the cardiovascular system and contributes to the formation of atherosclerotic lesions that damage the structure of blood vessels, making them more susceptible to deformation [4,5]. A phenomenon that is believed to initiate aneurysmal changes is the accumulation of fatty deposits, leading to an excessive load on the walls, which, in turn, hardens them and induces hypoxia [17]. Stackelberg et al., in a study based on a Swedish population, found that the risk of AAA was 30% higher in individuals with increased waist circumference compared to those with normal-size waist. It was also demonstrated that the risk of AAA increased by 15% with an increase in waist circumference by each 5 cm [18]. Golledge et al. also noted a positive correlation between anthropometric indicators of obesity, such as waist circumference and the waist–hip ratio, and the size of aortic dilatation. They argued that this relationship is particularly evident in cases where the extension exceeds 40 mm.

The excess of adipose tissue is associated with moderate inflammation state, and it is the central factor of metabolic syndrome which is connected with additional health problems such as insulin resistance, diabetes, and hypertension. Until recently, adipose tissue was considered an energy store in the form of triacylglycerols. It is now viewed as a tissue serving an endocrine function as it synthesizes and releases adipocytokines. Among these compounds, the most noteworthy are: adiponectin, leptin, resistin, interleukin 6 (IL-6), tumor necrosis factor alpha (TNF-α), apelin and dipeptidyl peptidase-4. Adipocytokines released from adipose tissue play an important role in the inflammatory process. Obesity-related inflammation is associated with increased macrophage infiltration, increased cytokine expression in adipose tissue and increased angiotensin II levels, which may contribute to AAA formation [19]. Since aneurysms are an inflammatory disease, therefore, PVAT is of particular interest as it is perivascular adipose tissue, and the adipocytokines released from it may play a direct role in aortic pathology [6,20]. It has been shown that the amount of PVAT is higher in the aortas of AAA patients than in the control group [6,21]. Moreover, PVAT from smokers (smoking is a major risk factor for AAA) has been found to show increased monocyte recruitment and higher ceramide levels (which triggers a sterile inflammatory reaction) than PVAT in non-smokers [6,22,23]. The importance of PVAT in aneurysm formation has been demonstrated in studies which indicated that PVAT deficiency reduces macrophage infiltration and attenuates AAA development [24,25]. The tissue is perceived as a paracrine and endocrine organ secreting inflammatory cytokines and adipokines. One of the key adipokines secreted by adipose tissue is adiponectin. It is an anti-inflammatory compound that is downregulated in obesity. This compound positively affects glucose and lipid metabolism [26]. It has been repeatedly shown that the plasma level of adiponectin is negatively correlated with the content of adipose tissue. In addition, in both human and animal studies, there was observed a strong negative correlation between the content of ceramides in adipose tissue and plasma adiponectin concentration, suggesting that the accumulation of ceramide in adipose tissue inhibits expression/secretion adiponectin [27,28,29].

Literature data show that in the walls of blood vessels, adiponectin inhibits the adhesion of monocytes (by lowering the expression of adhesion molecules) and inhibits the transformation of macrophages into foam cells. Experimental studies demonstrated that angiotensin II infusion in apolipoprotein E/adiponectin double-knockout (Apoe^−/−^ Apn^−/−^) mice led to an increased incidence of AAA as compared to Apoe^−/−^ mice. The results proved that adiponectin deficiency increased the infiltration of macrophages, increased the expression of pro-inflammatory cytokines, and elevated the activity of MMP-2 and MMP-9 in the dilated aortic wall [30]. Furthermore, studies in an animal AAA model have shown that adiponectin infusion reduces perivascular inflammation, prevents vascular infiltration of macrophage and effectively inhibits AAA development [30]. Subsequent studies in aneurysm induced mice fed a high-fat diet and intravenously administered adenoviral vectors encoding adiponectin (leading to an increase in adiponectin levels) also demonstrated a positive role of adiponectin in preventing AAA development [31]. The adiponectin-mediated inhibition of AAA development has been shown to occur by inhibiting the expression of pro-inflammatory cytokines, limiting aortic infiltration of inflammatory cells and thus reducing inflammation in adipose tissue [30,31]. In addition, adiponectin increases the production of nitric oxide (NO) in endothelial cells and stimulates angiogenesis [32].

So far, adiponectin is the only compound secreted by adipose tissue that inhibits AAA development. Other known active compounds, secreted by adipose tissue–inflammatory cytokines, have an opposite effect. Previous research has suggested a link between leptin and resistin concentration and the development of AAA. Leptin, which is secreted by adipose tissue, can also be released by macrophages that infiltrate PVAT. It has been shown experimentally that the periaortic administration of recombinant leptin to Apoe^−/−^ mice promotes the digestion of the extracellular matrix and the expansion of the aortic wall aneurysm [33]. Another compound secreted by adipose tissue as well as monocytes/macrophages that are perceived to contribute to aneurysms is resistin [5]. Studies in an experimental mouse aortic aneurysm model (Apoe^−/−^ mice with angiotensin II infusion) have shown that silencing the gene encoding a resistin-like molecule-beta inhibits the formation of aortic aneurysm. These changes were accompanied by a decrease in the accumulation of macrophages, a decrease in the expression of pro-inflammatory cytokines as well as MMP-2 and MMP-9 in the aortic wall [34]. Clinical studies revealed that plasma leptin and resistin concentration as well as greater amounts of PVAT were significantly higher in people with AAA compared to healthy counterparts [20].

The inflammatory process accompanying aneurysm lesions, also observed in the course of metabolic syndrome, boosts the progression of these lesions [1]. This is likely due to the fact that inflammation promotes the expression and/or activity of matrix metalloproteinases, which are very important factors in the formation of aneurysms.

## 4. Matrix Metalloproteinase in AAA

Aneurysms are characterized by the dilatation of arteries, wide walls, and a significant decrease in the elastin/collagen ratio [35,36,37,38,39]. These alterations are associated with inflammatory processes [35,40,41] and increased level of MMPs that regulate broad matrix destruction [35]. In the early stages of an abdominal aortic aneurysms development, matrix metalloproteinase-2 is the major elastase. Activation of this enzyme is mediated by membrane-type 1 matrix metalloproteinase (MT1-MMP), and its inhibition is mediated by tissue inhibitor of metalloproteinases type 2 (TIMP-2) [35]. MMP-2 is present both in healthy and aneurysmal tissues in conjunction with MT1-MMP and TIMP-2, and it exhibits target selectivity for elastin and fibrillar collagen [35,42,43]. It has been found that MMP-2 from abdominal aortic aneurysm vascular smooth muscle cells has three times higher levels than in cells taken from tissues with atherosclerosis [35,44,45]. Moreover, in clinical trials, it has been shown that in the inferior mesenteric vein of AAA patients, the MMP-2/TIMP-2 ratio was significantly higher than in the control group. These data imply that the vessels of AAA patients have a systemic trend to enhanced proteolysis. A histological analysis confirmed that this trend indicated the degradation of elastin fibers inside abdominal aortic aneurysm and a significant reduction in elastin within the media (this phenomenon was not observed in the control group) [32]. It follows that MMP-2 overexpression may be associated with early elastolysis seen in minor aneurysms, and it is observed not only within the aneurysm but in arteries and veins distant from its location [35]. MMP-2 is the primary metalloproteinase in minor aneurysms, whereas MMP-9 is involved in late aneurysm development [35,46,47]. Literature data indicated that MMP-9 clearance depends on the expression of low-density lipoprotein receptor-1 (LRP1) -bound protein, therefore, LPR1 is considered an important element in the pathogenesis of abdominal aortic aneurysm [48,49]. It is a cell-surface receptor found in numerous organs, e.g., liver, cerebrum, intestine, and muscle, that transmits many intracellular signaling pathways involved in the regulation of inflammation and tissue remodeling [48,50,51,52]. It may connect to a number of agonists for endocytosis [48,53,54,55,56,57]. LRP1 has been reported to serve as a scavenger of pericellular MMP-9 [48,58,59,60,61,62]. Available data suggest that LRP1 depletion reduces the uptake and destruction of MMP-9 in the fetal fibroblast cell line [48,58]. In addition, LRP1 gene silencing in VSMC also significantly decreased the clearance of MMP-9. It follows that lowering the level of LRP1 in VSMC may impede the removal of pericellular MMP-9, leading to an excess of MMP-9 remaining in the extracellular matrix [48]. In addition, it has been shown that LRP1 downregulation is tightly regulated by microRNA-205 by inhibiting translation in human VSMCs [48]. Therefore, LRP1 activity appears to play an important role in the pathogenesis of an abdominal aortic aneurysm, since LRP1 expressed in vascular smooth muscle cells may influence the concentration of MMP-9 in the extracellular matrix of the aortic wall, potentially controlling elastin proteolysis and aneurysm development [48].

## 5. Sphingolipids Implication in the Pathogenesis of Abdominal Aortic Aneurysms

Sphingolipids are a group of lipids found in all eukaryotic cells. A central molecule in the sphingolipid metabolism is ceramide. This compound is involved in the regulation of many cellular processes including proliferation, differentiation, migration, adherence, aging, cell death (e.g., necrosis, apoptosis) and inflammation [6,63]. Sphingosine-1-phosphate, a ceramide derivative, is a key mediator in the processes of lymphocyte and mast cell migration, angiogenesis, and the differentiation of endothelial cells and smooth muscles. It has a significant impact on the adhesion and survival of cells, which, inter alia, lead to ischemia–reperfusion injury that causes changes in blood vessels [64]. A rheostat of ceramide and S1P determines the fate of cells, elevated levels of ceramide and sphingosine lead to cell death, while a boosted content of S1P increases cell survival [65].

**Figure 2:**
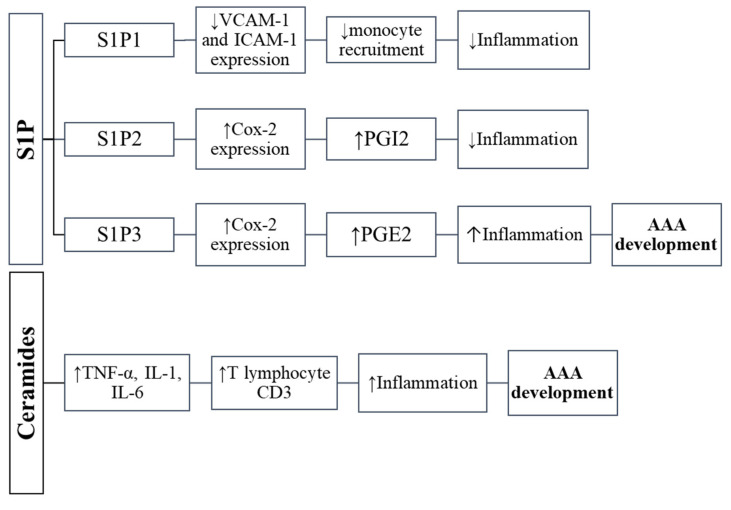
The influence of sphingolipids on inflammation in abdominal aortic aneurysm.

Ceramide and sphingomyelin have been shown to contribute to the formation of atherosclerosis, which often coexists with AAA [6,66,67]. Moreover, the content of sphingolipids increases in the state of obesity [6,68]. Ceramide has been found to be associated with signaling for pro-inflammatory mediators (e.g., tumor necrosis factor alpha, IL-1 and IL-6) (Figure 2). People with obesity develop inflammation, which is characterized by an increase in the number of macrophages infiltrating adipose tissue and elevated expression and secretion of inflammatory cytokines such as TNF-α, IL-6 and IL-1β. Some of these cytokines have been shown to be implicated in the production of ceramides [69]. It has been shown that the level of TNF-α in both adipose tissue and serum is elevated in people with obesity and positively correlates with the ceramides content [70]. In cultured cells, TNF-α has been demonstrated to stimulate ceramide accumulation by regulating the expression of genes encoding enzymes responsible for the formation of ceramide, i.e., serine palmitoyltransferase and the sphingomyelinase—an enzyme that catalyzes the hydrolysis of sphingomyelin [69,71,72]. In addition, inflammatory cytokines such as IL-6 and TNF-α can also alter the expression and activity of MMPs and TIMPs [73]. It has been demonstrated that TNF-α induces the inflammatory reaction via ceramides [6,74], and MMP-2, which can be produced and/or activated by macrophages, contributes to the ceramide synthesis and activation [6,75,76]. It has been also shown that the expression of MMP-9 is up-regulated by ceramide [77,78,79]. On the other hand, adiponectin, also synthesized in adipose tissue, an anti-inflammatory molecule that has a protective effect on aneurysm formation, is likely to have a broad spectrum of activity by lowering the level of ceramides. It has been found that adiponectin receptors (AdipoR) exhibit ceramidase activity, which is an enzyme that breaks down ceramide into sphingosine and fatty acid [80].

Other ways by which ceramide can affect AAA formation are increased ROS production and the induction of apoptosis in VSMC. Vascular smooth muscle apoptosis is one of the basic features of aneurysms. Ceramide has been shown to induce apoptosis in different cell types, including VSMC [81,82,83]. It has been shown that ROS play an important role in ceramide-mediated apoptosis. On the other hand, ROS induce the production of ceramides by activating ceramide-producing enzymes, which leads to apoptosis, while inhibiting the production of S1P, which promotes survival [84,85].

Recently, the attention of researchers has also focused on the implication of ceramide-1-phosphate (C1P) in the regulation of inflammatory processes. C1P is a bioactive lipid that is formed by the phosphorylation of ceramide. This reaction is catalyzed by ceramide kinase (CerK). C1P, unlike ceramide, is an anti-apoptotic molecule, but its role in the regulation of inflammatory processes is ambiguous. The compound exhibits both pro-inflammatory and anti-inflammatory properties depending on the cell type [13,86,87]. On the one hand, studies have shown that C1P is a potent stimulator of cytosolic phospholipase A2 (cPLA2) with the subsequent release of arachidonic acid and prostaglandin biosynthesis, which placed C1P on the list of pro-inflammatory compounds. Moreover, other research showed that the deletion of CerK suppresses obesity-related inflammatory cytokines IL-6 and TNFα and reduced macrophage infiltration in adipose tissue, resulting in reduced inflammatory responses in animals fed a high-fat diet [88,89]. Surprisingly, CerK -/- animals still have a significant amount of C1P, indicating that there may be alternative pathways leading to the formation of C1P [13]. On the other hand,, C1P has been found to inhibit both acute and chronic inflammation caused by smoking. This is an important issue in terms of aneurysms pathogenesis, as smoking is one of the major risk factors for AAA formation [90]. The anti-inflammatory effect of C1P in this case was associated with the inhibition of the expression of pro-inflammatory cytokines: TNFα, IL-1β, IL-6, and macrophage inflammatory protein-2 (MIP-2) in epithelial cells and neutrophils [90]. However, it has also been shown that in macrophages, exogenous C1P increases the expression of the two most important metalloproteinases in the development of aneurysms: MMP-2 and MMP-9, but so far, no studies have been conducted that would directly confirm the involvement of C1P in the formation of aneurysms [91].

## 6. Sphingosine-1-Phosphate

Sphingosine-1-phosphate is a bioactive sphingolipid produced by the phosphorylation of sphingosine, which is catalyzed by two sphingosine kinase isoenzymes (SphK1 and SphK2) [11,92] (Figure 1). In plasma, platelets are the primary source of S1P, but other cell types such as neutrophils, erythrocytes and mononuclear cells can also produce it [11,92,93]. S1P is a key regulator in vascular inflammation. It has been shown that the concentration of S1P in the blood is lower in individuals with abdominal aortic aneurysm, and there was observed a statistically significant negative correlation between the occurrence of AAA and the level of S1P in the serum [94,95]. S1P triggers inflammation by interacting with five distinct receptor types (S1PR): 1, 2, 3, 4, and 5. They belong to the G protein-coupled receptor family. Differential S1PR expression is considered to have an effect on cell survival and death [11,96] as well as pathological processes including inflammatory reaction, carcinogenesis, and immunological modulation [11,92,97,98,99]. The main S1P receptor in the vascular system is S1PR2; however, S1PR1 and S1PR3 are also found in both endothelial cells and vascular smooth muscle but in much smaller amounts [11,100,101]. The distinctive expression of S1PR has been shown to either promote or prevent inflammatory infiltration in a variety of cell types by activating cyclooxygenase 2 (COX-2) expression [11,98], which is followed by prostacyclin (PGI2) or prostaglandin E2 (PGE2) synthesis [11,102,103,104] (Figure 2). Since inflammation is the basis of AAA formation, and both S1P and S1PRs are important regulators of inflammation in blood vessels, it can be assumed that S1P and S1P-dependent signaling pathways may contribute to the pathogenesis of the aneurysm. Recent studies have proven the importance of S1PR in the pathogenesis of AAA [11,105]. In the abdominal aortic aneurysm, it was shown that the level of S1PR2 decreases, while the amount of S1PR3 increases compared to the control tissue. In contrast, the S1PR1 was absent in neither the AAA aorta nor in the control [11]. In reply to sphingosine-1-phosphate, S1PR2 can induce COX-2 expression and synthesize prostacyclin (PGI2) [11,104,106,107]. PGI2 reveals anti-inflammatory properties as well as the ability to relax vascular smooth muscle cells [11,108]. Therefore, the reduced expression of anti-inflammatory S1PR2 in abdominal aortic aneurysm vascular smooth muscle cells may impede prostacyclin synthesis and, as a result, lead to severe inflammatory reaction in AAA [11,109]. The S1PR3 promotes inflammatory reactions by inducing COX-2 expression and subsequent PGE2 synthesis in a variety of cell subtypes [11,102,103,110,111]. Through chemotaxis and PGE2 production, S1PR3 may play a role in the severe inflammation observed in the course of abdominal aortic aneurysms and atherosclerosis [11].

In addition, high blood levels of high-density lipoproteins (HDL) have been shown to be associated with a lower risk of an abdominal aortic aneurysm enlargement. The mechanism by which HDL protects against aneurysms has not yet been elucidated. However, interestingly, HDL is an important carrier of S1P, and it appears that this positive effect of HDL is, at least in part, dependent on the signaling pathways in which S1P is involved [94,112]. It has been demonstrated that S1P activates numerous second messenger systems containing serine/threonine protein kinase Akt [94,113], and it inhibits inflammation in endothelial cells (ECs) and VSMCs [94,114]. Serine/threonine protein kinase regulates several cell signals, including those controlling the inflammatory reaction and endothelial nitric oxide synthase (eNOS), which may be involved in the development of abdominal aortic aneurysm and atherosclerosis. S1P activates eNOS via Akt/phosphoinositide 3-kinase-dependent and calcium-dependent pathways, resulting in NO generation [94,115,116]. Moreover, there are also reports of fenofibrates, drugs prescribed for the treatment of hypertriglyceridemia and mixed dyslipidemia, whose lipid-altering effects are mediated through activation of PPAR-α (peroxisome proliferator-activated receptor-α). Fenofibrates are pleotropic, lowering fibrinogen and CRP levels and enhancing flow-mediated dilatation of vessels [117]. It has been found that fenofibrate recipients had higher blood HDL levels, serum S1P concentrations, as well as boosted aortic Akt1 and eNOS activity as compared to control group [117]. Since HDL is a major carrier of S1P in the blood, the protective role of fenofibrate may be mediated by HDL-bound sphingosine-1-phosphate and may arise from the activation of S1P receptors via HDL-S1P and downstream signaling pathways including the Akt-eNOS pathway. Aneurysm-induced mice treated with fenofibrate have demonstrated a reduced immune cell infiltration, increased activity of the enzymes eNOS and iNOS, and increased levels of receptors (S1PR1 and S1PR3) [94]. These data suggest that the effects of fenofibrates are mediated by S1P.

## 7. Conclusions

The attributes of abdominal aortic aneurysm pathogenesis include, in addition to inflammatory processes, the production of reactive oxygen species and the programmed cell death of vascular smooth muscle cells [2]. Abdominal aortic aneurysms are more common than thoracic aneurysms and more often need surgical therapy [2,118,119]. Increasing evidence points to the potential influence of obesity and the related inflammation, manifested by increased infiltration of adipose tissue by macrophages (which are an important source of cytokines and MMPs), increased expression of pro-inflammatory cytokines in adipose tissue, including PVAT, and impaired sphingolipid metabolism on the development of an abdominal aortic aneurysms [19]. It has been found that pro-inflammatory cytokines, including resistin, leptin, and TNFα promote the digestion of the extracellular matrix by MMP-2 and MMP-9, leading to the formation of aneurysms, while adiponectin is the only known compound that is secreted by adipose tissue and limits the development of aneurysms. It is an anti-inflammatory adipokines that is downregulated in obesity. Experimental studies have shown that the infusion of adiponectin in an animal AAA model reduces inflammation, limits macrophage infiltration and is effective in inhibiting AAA development [30,31]. It seems that at least some of the broad-spectrum activity of adiponectin is achieved by lowering the level of ceramides. Adiponectin receptors (AdipoR) have been shown to exhibit the activity of ceramidase, which is an enzyme that catalyzes the breakdown of ceramide into sphingosine and a fatty acid [80]. Obesity has been shown to be associated with altered sphingolipid metabolism. Among sphingolipids, there are compounds that play an opposite role in the cell: ceramide is a pro-apoptotic compound that also mediates the development of inflammation, while sphingosine-1-phosphate has a pro-proliferative and anti-inflammatory effect. Ceramide and sphingomyelin may contribute to atherosclerosis associated with the pathogenesis of AAA [6,66,67]. Moreover, the increased level of ceramide observed in obesity is associated with a decrease in the concentration of adiponectin, an increase in the secretion of pro-inflammatory cytokines and the up-regulation of MMP-9 [6,63]. Furthermore, ceramide increases the production of ROS, which increases oxidative stress in many cells, including VSMC cells and contributes to their apoptosis. On the other hand, the inhibition of ROS-generating enzymes or treatment with antioxidants inhibits sphingomyelinase activation and ceramide production [10]. All these factors promoted by the increased level of ceramide contribute to the formation of aneurysms. However, another sphingolipid, S1P, is an important regulator of inflammation in blood vessels. Blood levels of S1P have been shown to be lower in people with abdominal aortic aneurysm as compared to their healthy counterparts [94,95]. S1P acts through five types of membrane receptors: S1PR1–5. The main S1P receptors in the vascular system are S1PR2, S1PR1 and S1PR3 [11,100,101]. The distinctive expression of S1PR has been shown to either promote or prevent inflammatory [11,98,102,103,104]. The literature data indicate that the S1PR2-mediated action of S1P has an anti-inflammatory effect, while the activation of S1PR3 initiates the pro-inflammatory pathway [11,108,109]. In addition, high blood levels of HDL have been shown to play a protective role against an abdominal aortic aneurysm enlargement. Since HDL is an important carrier of S1P, it appears that this positive effect of HDL depends on the signaling pathways in which S1P is involved.

## Data Availability

Not applicable.
